# Commitment Lotteries Promote Physical Activity Among Overweight Adults—A Cluster Randomized Trial

**DOI:** 10.1093/abm/kax017

**Published:** 2018-01-25

**Authors:** Koen van der Swaluw, Mattijs S Lambooij, Jolanda J P Mathijssen, Maarten Schipper, Marcel Zeelenberg, Stef Berkhout, Johan J Polder, Henriëtte M Prast

**Affiliations:** 1 Tilburg University, Tranzo Scientific Center for Care and Welfare, Tilburg School of Social and Behavioral Sciences, LE Tilburg, The Netherlands; 2 National Institute of Public Health and the Environment (RIVM), Department of Quality of Care and Health Economics, Center for Nutrition, Prevention and Health Services, BA Bilthoven, The Netherlands; 3 National Institute of Public Health and the Environment (RIVM), Department of Statistics, Informatics and Modelling, Center for Nutrition, Prevention and Health Services, BA Bilthoven, The Netherlands; 4 Tilburg University, Department of Social Psychology, Tilburg School of Social and Behavioral Sciences, LE Tilburg, The Netherlands; 5 VU Amsterdam, Department of Marketing, School of Business and Economics, HV Amsterdam, The Netherlands; 6 High Five Health Promotion, Department of Quality Management, Schinkeldijkje, CE Aalsmeer, The Netherlands; 7 Tilburg University, Department of Finance, Tilburg School of Economics and Management, LE Tilburg, The Netherlands

**Keywords:** Behavior change, Physical activity, Health promotion, Commitment devices, Behavioral economics, Deadlines

## Abstract

**Background:**

The World Health Organization has identified physical inactivity as the fourth leading risk factor for global mortality. People often intend to engage in physical activity on a regular basis, but have trouble doing so. To realize their health goals, people can voluntarily accept deadlines with consequences that restrict undesired future behaviors (i.e., commitment devices).

**Purpose:**

We examined if lottery-based deadlines that leverage regret aversion would help overweight individuals in attaining their goal of attending their gym twice per week. At each deadline a lottery winner was drawn from all participants. The winners were only eligible for their prize if they attained their gym-attendance goals. Importantly, nonattending lottery winners were informed about their forgone prize. The promise of this counterfactual feedback was designed to evoke anticipated regret and emphasize the deadlines.

**Methods:**

Six corporate gyms with a total of 163 overweight participants were randomized to one of three arms. We compared (i) weekly short-term lotteries for 13 weeks; (ii) the same short-term lotteries in combination with an additional long-term lottery after 26 weeks; and (iii) a control arm without lotteries.

**Results:**

After 13 weeks, participants in the lottery arms attained their attendance goals more often than participants in the control arm. After 26 weeks, we observe a decline in goal attainment in the short-term lottery arm and the highest goal attainment in the long-term lottery arm.

**Conclusions:**

With novel applications, the current research adds to a growing body of research that demonstrates the effectiveness of commitment devices in closing the gap between health goals and behavior.

**Clinical Trial information:**

This trial is registered in the Dutch Trial Register. Identifier: NTR5559

## Teaser

Psychologically designed lottery-deadlines promoted gym attendance among overweight adults. The combination of weekly commitment lotteries and an additional long-term lottery yielded the most durable effects.

## Introduction

Physical activity (PA) is a key behavioral determinant of individual and public health [1, 2]. Regular PA contributes to cardiovascular fitness and weight management, and reduces the risks of, among others, cardiovascular disease, cancers, diabetes mellitus type 2, and obesity [3–5]. Consequently, the World Health Organization and governments worldwide recommend citizens to exercise on a regular basis [1, 6, 7]. Despite ample endorsements and many intentions to lose weight and exercise regularly [8, 9], 79% of Americans and 66% of Europeans do not meet recommended levels of PA [10, 11]. Likewise, 74% of Americans and 62% of Europeans are overweight (body mass index [BMI] ≥ 25) [12, 13].

Although people often intend to change their behavior and engage in PA on a regular basis, they systematically fail to do so [14, 15]. Behavioral economics, operating at the intersection of economics and psychology [16], provides insights that help to explain the difficulties of behavior change, including present bias: the human tendency to disproportionally overweigh costs and benefits that are immediate over those that are delayed [17–19]. Correspondingly, long-term health goals are widely adopted, but are mostly not fully achieved [9, 14, 20]: despite previous intentions, the immediate costs (e.g., exercising) overshadow the delayed benefits (e.g., good health), resulting in procrastination [21].

To not fall prey to this pattern, people can voluntarily accept meaningful deadlines that impose potential costs on undesired future behaviors, known as commitment devices [21, 22]. A common application of a commitment device is the “deposit contract,” where individuals voluntary deposit money that they will lose if they fail to achieve a predetermined personal goal at a deadline [22–24]. By restricting behavior ahead of time, commitment devices strategically avert present-biased tendencies and can hereby help individuals in conserving their intended exercising behavior [25].

Although physical inactivity is hazardous in all BMI ranges [2, 26], overweight (BMI ≥ 25) and obese (BMI ≥ 30) individuals are especially likely to benefit from commitment devices for PA because they generally exercise less than normal-weight individuals [27], while regular PA can contribute to weight loss and management. Besides, overweight and obesity have been associated with a relatively strong disposition to overweigh the present over the future (i.e., present bias) [28–31] and commitment devices are designed to preempt this.

Drawing on previous applications of behavioral economics in supporting health behavior change [32], we tested multiple lottery deadlines intended to help overweight adults in attaining their gym-attendance goals. Research suggests that people are generally regret averse, meaning that they anticipate regret and often make decisions that minimize regret in the future [33]. The lottery deadlines were designed to leverage regret aversion by incorporating a key feature of the Dutch postal code lottery (2.5 million players per drawing). In the postal code lottery all postal codes can win, but only the residents who purchased tickets get a prize. Inevitably, residents of the winning region who did not purchase tickets discover that they would have had a prize if they had decided differently in the past. Accordingly, regret aversion has been found to motivate the decision to purchase lottery tickets [34].

In the present study, participants committed to their goal of attending their gym twice per week by voluntarily accepting multiple lottery deadlines. At each lottery deadline a winner was drawn from all participants. The winners, however, were only eligible to receive their prize if they attained their gym-attendance goals. Importantly, lottery winners who did not attain their goal were informed about their forgone prize. The promise of feedback on “what would have been” was designed to emphasize the possibility of regret at the deadlines [35].

We set up a three-arm cluster randomized trial across six gyms to examine if commitment lotteries would support overweight adults in attaining their goal of attending their gym twice per week. We compared (i) weekly short-term lotteries for 13 weeks; (ii) the same short-term lotteries in combination with an additional long-term lottery after 26 weeks; and (iii) a control arm without lotteries. We examined the effect of the lottery interventions on weekly individual goal attainment over 13, 26, and 52 weeks compared to a control arm. This article reports on the results after 13 and 26 weeks.

We hypothesized that after 13 weeks, participants in both lottery arms would be more likely to attain their week goals than participants in the control arm. Behavioral economic commitment schemes generally result in behavior change in the short run, but the changes are mostly not fully maintained [36–38]. Therefore, we expanded the short-term deadlines with an additional long-term deadline to test if this would promote long-term goal attainment. Hence, after 26 weeks, we expected a decline in goal attainment in the short-term lottery arm and the highest goal attainment in the long-term lottery arm [39].

## Method

### Design

**Fig. 1.**
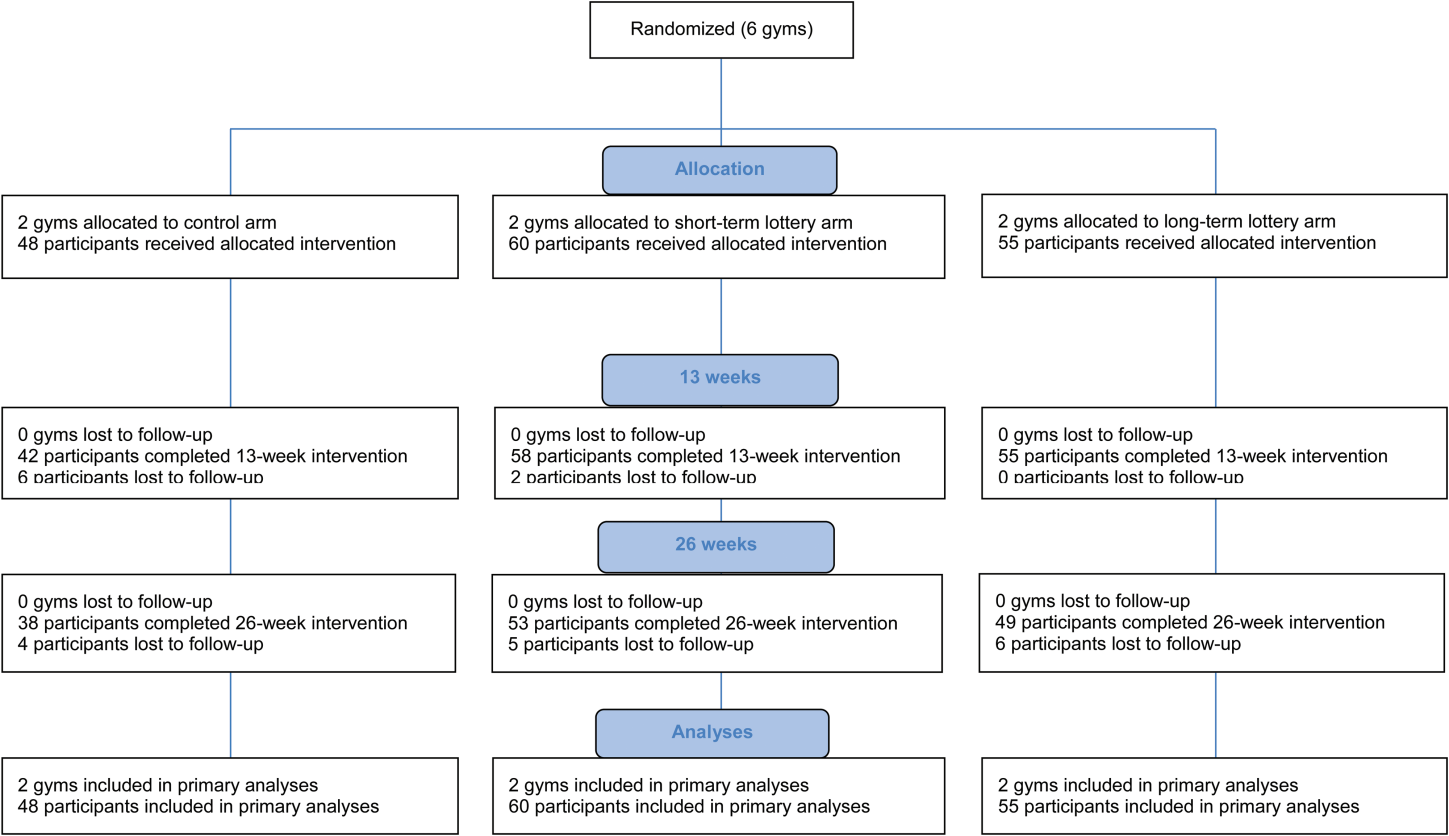
Study design and flow of gyms and participants.

The rationale and protocol of this trial have been published elsewhere [39]. The design is a three-arm, parallel group, cluster randomized trial running for 52 weeks with 163 participants in six corporate gyms (clusters) across the Netherlands. Figure 1 displays the study design and flow. The trial protocol and materials were reviewed and approved by the Tilburg University Ethical Review Board (EC-2014.42a). The study is registered in the Dutch Trial Register (NTR5559) and lottery drawings were performed by the independent Game Management Department of the Dutch State Lottery under supervision of a notary.

### Participants & Enrollment

Gyms were eligible to participate if the managers expressed their interest in scientific research prior to randomization. The six gyms were a random convenience sample from 36 corporate gym sites across the Netherlands hosted by fitness enterprise High Five. Next to written information and an oral briefing, gyms received a tailored video containing the rationale and protocol of the trial. With a standardized recruiting text, provided to the gyms, gym managers recruited new and existing members who were looking for a commitment device for regular exercise, via e-mail, company web pages, and in person. The material summarized the nature and procedure of the study and directed candidates to the gym personnel. We aimed to recruit a minimum of 25 participants per gym, but allowed gyms to screen more participants.

Candidates were eligible if they explicitly stated to have the goal to exercise twice or more per week, were overweight (BMI ≥ 25 < 40), between the age of 18–65, and had not planned a leave of absence of more than 4 weeks in the first 26 weeks of the trial. Together with the gym personnel, candidates weighed on a provided scale (KERN; 0.1% precision) and filled out a digital questionnaire which immediately identified whether the candidate was eligible or not. After providing informed consent, applicants were entered into the study.

### Interventions

This trial compares two intervention arms to one control arm. The interventions pertain to the participant level. The American College of Sports Medicine and the American Heart Association endorse vigorous exercise for 20 min, 3 days a week, and muscular strength and endurance training 2 days a week [1]. Consequently, setting the goal of attending the gym 2 days a week was considered beneficial, while challenging but attainable [39]. Therefore, participants in all three arms set the goal to attend their gym twice per week (the week goal) and were handed a randomly generated three-digit study ID prior to the start of the trial. Upon entering their gym, all participants were required to register their attendance with their study ID on trial iPads, provided to the gyms. All participants were offered a monthly overview of their attendance via e-mail.

#### Intervention arm 1: short-term lottery

For 13 weeks, participants in this arm participated in a free weekly lottery worth €100 each drawing. The winning number (study ID) was drawn from all participants in this arm (participants knew that they could always win the lottery) and communicated to all via text message and e-mail (participants knew that they would always learn the outcome). The winners only received their prize if they attended their gym at least twice that week (the week goal). Importantly, lottery winners who did not attain their week goal were informed about their forgone prize. All other participants knew whether the week prize was awarded or not, but not to whom. Notably, every new week offered a new opportunity to win and to keep attaining exercise goals, regardless of prior success. This feature facilitates the human inclination to use temporal landmarks (e.g., Mondays) as a fresh start by relegating misfortune to the past [40]. The weekly expected monetary value for a fully compliant subject was 1/60 = €1.67. Note, however, that the lotteries were designed to emphasize the deadlines and not as a payment.

#### Intervention arm 2: long-term lottery

The intervention in this arm was identical to the short-term lottery arm in the first 13 weeks. The weekly expected monetary value for a fully compliant subject was 1/56 = €1.78. Additionally, Weeks 14–26 were also part of the intervention (participants knew this prior to the start of the trial). After Week 26, a luxury vacation cheque for the winner and four friends or family members (communicated as such to participants, worth €5,400) was awarded. The winning number was again drawn from all participants and communicated to all via text message and e-mail. Participants were informed that the prize could only be claimed if the winner would attain the week goal in at least 9 of the second 13 weeks (70% between Weeks 14 and 26). Because Weeks 14–26 fell in the national holiday season, the 9:13 success ratio provided participants the opportunity to enjoy a vacation and still be eligible for their prize. Participants knew that if the winner would not meet the requirements for obtaining the prize, he or she would receive a small consolation prize and another number would be drawn until the prize could be claimed.

#### Control arm

In the control arm, participants also set the goal to attend the gym twice per week and were monitored in their attendance and secondary outcomes, but no commitment devices were offered. As such, the lotteries were the only designed differences between control and intervention arms. Participants in the control arm were also offered monthly statistics on their performance via e-mail.

### Outcomes and Measures

The primary outcome of interest was goal attainment (week gym attendance ≥ 2) measured at the participant level and assessed by requiring participants to check in to the trial iPad when entering their gym. Baseline attendance levels, nationality, age, sex, education, and income level were assessed via questionnaires and are displayed in Table 1.

**Table 1:** Baseline Participant Characteristics Displayed by Study Arm

Characteristic	Control (*n* = 48)	Short-term lotteries (*n* = 60)	Long-term lottery (*n* = 55)
Age, mean (*SD*)	50 (9.84)	49.3 (9.33)	45 (9.58)
Gender, no. (%)			
Female	16 (33.3)	21 (35)	13 (23.6)
Male	32 (66.7)	39 (65)	42 (76.4)
No survey response, no. (%)	3 (6.25)	0 (0)	1 (1.67)
Nationality, no. (%)			
Dutch	36 (80)	52 (86.7)	52 (96.3)
Other	12 (20)	8 (13.3)	3 (3.7)
Education, no. (%)			
Pre-vocational education	3 (7.9)	7 (11.5)	4 (7.4)
Pre-university education	3 (6.7)	2 (3.3)	10 (18.5)
Senior vocational training	11 (24.4)	20 (33.3)	5 (9.3)
Vocational colleges	19 (42.2)	15 (25)	23 (42.6)
University education	9 (20)	15 (25)	10 (18.5)
Other	0 (0)	1 (1.7)	2 (3.7)
Monthly net income, no. (%)			
<€1,000	0 (0)	0 (0)	1 (1.8)
€1,000 to €2,000	10 (20.8)	6 (10)	3 (5.5)
€2,000 to €3,000	19 (39.6)	32 (53.3)	24 (43.6)
€3,000 to €4,000	8 (16.7)	15 (25)	19 (34.5)
€4,000 to €5,000	2 (4.2)	1 (1.7)	2 (3.6)
€5,000 tot €6,000	0 (0)	2 (3.3)	1 (1.8)
>€6,000	1 (2.1)	0 (0)	0 (0)
Did not wish to answer	5 (10.4)	4 (6.7)	4 (7.3)
Baseline gym attendance^a^, mean (*SD*)	1.82 (0.88)	1.46 (1.17)	1.55 (1.04)
Weight, mean (*SD*)	90.14 (14.38)	96.12 (14.12)	96.6 (13.94)
Fat percentage, mean (*SD*)	33.78 (6.32)	35.52 (7.54)	36.83 (9.22)
BMI, mean (*SD*)	28.9 (3.20)	30.4 (3.73)	30.19 (3.47)
Obese, no.(%)	13 (27.1)	23 (38.3)	26 (47.3)

BMI = body mass index.

^a^Participants answered the question; “On average, how often per week did you attend the gym in the last two months?”.

### Sample Size and Randomization

The sample size calculation for this trial has been reported in detail before [39]. Anticipating a 0.35 difference between proportions, based on meta-analysis by Haff et al. [32], and accounting for the clustered design, we estimated a required sample size of 36 per arm and aimed to include at least 50 participants per arm, allowing for 25%–30% attrition. No within-gym randomization was performed to avoid intervention contamination, maintain blinding at the participant level, and to minimize the administrative burden for the gym personnel. Therefore, every trial arm included two gyms. Participants were informed that there were two gyms in their arm, but not about the content of the interventions in the other gyms and arms.

Based on anonymized member data, we were able to distinguish three gyms with a relatively high proportion and three gyms with a relatively low proportion of overweight members. By computer generation, first high-proportion gyms and next low-proportion gyms were randomly allocated to one of three arms, preventing large differences in enrollment time.

### Statistical Methods

Analyses followed the intention-to-treat principle and were conducted in R version 3.3.1 and SPSS Statistics version 22 (IBM Corp, Armonk, NY) with statistical significance set at *p*<.05. Goal attainment was evaluated binary (0 = no, 1 = yes) at the participant level. Multivariate logistic mixed models were used to assess between-arm differences in goal attainment between Weeks 1–13 and 14–26 controlled for baseline PA, age, and sex. The control arm was modeled as the reference category and gyms were modeled as random intercepts.

In the mixed models, intervention effects are adjusted for the dependence of the outcome within gyms and adjusted for baseline PA differences. As such, in estimating the coefficients, the mixed models account for the clustered data pattern. To further inspect within-gym effects, we additionally performed sensitivity analyses by excluding each gym from the models once and comparing effects from these models to effects in the complete model.

## Results

**Fig. 2.**
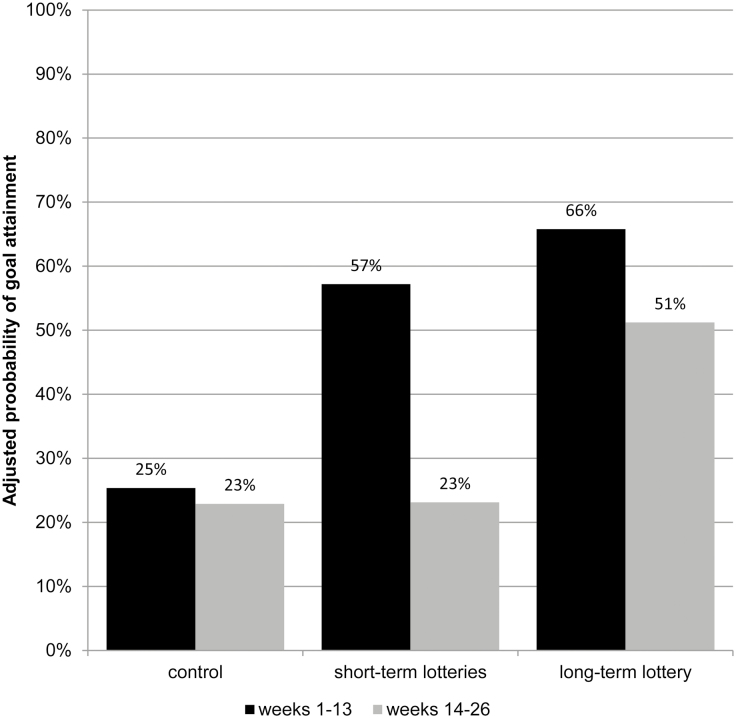
Adjusted probabilities of goal attainment (week attendance ≥ 2) between Weeks 1–13 and 14–26, displayed by trial arm.

Table 2 displays the average frequency of goal attainment per 13 weeks. Additionally, Fig. 2 displays the adjusted probabilities of goal attainment between Weeks 1–13 and 14–26 per arm.

**Table 2:** Average Frequency of Successful Weeks (Gym Attendance ≥ 2) per Study Period

	Weeks 1–13	Weeks 14–26	Weeks 1–26
	Mean (*SD*)	Mean (*SD*)	Mean (*SD*)
Study arm			
Control	3.54 (4.03)	3.38 (4.06)	6.92 (7.45)
Short-term lotteries	7.33 (3.58)	3.18 (3.37)	10.52 (6.20)
Long-term lottery	8.31 (4.05)	6.25 (4.38)	14.52 (7.84)

Adjusted for within-gym clustering, baseline attendance, age, and sex.

### Weeks 1–13

In both lottery arms, 8 of the 13 lottery winners (62%) received their prize. Participants in both lottery arms were more likely to attain their week goal than participants in the control arm. On average, participants in the control arm attained 27% of their week goals opposed to 55% and 63% in the short-term lottery and long-term lottery arm, respectively. Accordingly, the mixed logistic model (Table 3) showed a statistically significant intervention effect on goal attainment for the short-term lottery arm (odds ratio [OR] = 3.39; 95% CI, 1.20–12.92) and the long-term lottery arm (OR = 5.66; 95% CI, 1.72–18.66). The intervention effect did not differ significantly between both intervention arms (OR= 1.44; 95% CI, 0.44–4.70). The results of the sensitivity analyses were qualitatively similar to those based on primary analysis: the direction of effects in the sensitivity analyses did not diverge from the intervention effects in the complete model.

**Table 3:** Logistic Mixed Models Predicting Goal Attainment (Week Attendance ≥ 2) Between Weeks 1–13 and 14–26

	Weeks 1–13	Weeks 14–26
	Odds ratio (95% CI)	Odds ratio (95% CI)
Study arm		
Short-term lotteries	3.93* (1.20–12.92)	1.01 (0.37–2.80)
Long-term lottery	5.66** (1.72–18.66)	3.53* (1.28–9.77)
Participant characteristics		
Baseline attendance	1.28** (1.17–1.41)	1.40** (1.27–1.55)
Age	1.00 (0.99–1.01)	1.02** (1.01–1.03)
Male vs. female	0.54** (0.43–0.68)	0.73** (0.58–0.93)

Intracluster correlation (Weeks 1–13): 0.10, (Weeks 14–26): 0.07.

*Significant at *p* < .05; **Significant at *p* < .01.

### Weeks 14–26

On average, participants in the control arm and short-term arm attained 25% and 24% of their week goals, respectively, whereas participants in the long-term arm on average attained 48% of their week goals. Participants were eligible to receive the long-term lottery if they attained their goal in at least 9 of the second 13 weeks. In total, 55% of participants in the long-term lottery arm attained the week goal in ≥9 weeks. The mixed logistic model showed a statistically significant intervention effect on goal attainment for the long-term lottery (OR = 3.53; 95% CI, 1.28–9.77). Besides, participants in the long-term lottery arm were significantly more likely to attain their goals than participants in the short-term lottery arm (OR = 3.48; 95% CI, 1.27–9.57). In contrast to Weeks 1–13, the likelihood of goal attainment in the short-term lottery arm no longer differed significantly from the control arm (OR = 1.01; 95% CI, 0.37–2.80).

The sensitivity analyses showed qualitatively similar intervention effects for the long-term lottery arm. The estimated coefficient of the short-term lottery arm was sensitive to exclusion of gyms from the control arm. The non-effect in the complete model became a negative effect when excluding the least performing gym in the control arm from the analyses. The effect became positive when excluding the best performing control-gym from the analyses.

## Discussion

The results from this cluster randomized trial show that commitment lotteries can help overweight adults in attaining their goal of attending their gym twice per week. Participants who voluntarily committed to 13 weekly lottery deadlines were more likely to attain their goal of attending their gym twice per week than participants in the control arm. Furthermore, participants who were assigned to an additional lottery deadline after 26 weeks were more likely to attend their gym twice per week after 26 weeks than participants without this long-term lottery deadline.

Although this trial showed that weekly lotteries were effective in providing short-term commitment, goal attainment decreased in absence of an additional long-term deadline. As expected, the additional long-term lottery deadline partly averted the decline in PA after an initial period of success.

The present findings expand knowledge on the use of commitment devices to facilitate behavior change. The demand for commitment devices has been illustrated in an increasing body of behavioral research. For example, people voluntarily restrict future spending [41, 42], eating [43], or smoking [24] to facilitate (retirement) saving, weight loss, and quitting attempts. The present trial contributes with a novel behavioral context (gym attendance) and the application of a long-term lottery deadline.

To overcome present-biased decision-making and procrastination, behavioral research generally recommends increasing immediate (costs) benefits of (un)desirable behaviors as a strategy for behavior change [44, 45]. In this reasoning, the effectiveness of the weekly lottery deadlines can be explained by their ability to impose nearby consequences on procrastination. A nearby deadline with the chance to win, but miss out on €100 limits the time window for action and hereby prioritizes the desired behavior. Previous studies have used comparable strategies to effectively support medication adherence [46], weight loss [36], and walking [38].

In contrast to multiple nearby deadlines, a distant deadline interferes less with present-biased preferences and leaves more time for procrastination. This was demonstrated in research by Ariely and Wertenbroch [21] in which students’ academic performance decreased when they accepted one distant deadline opposed to multiple nearby deadlines. However, in the present trial, the long-term lottery deadline partly averted the decline in goal attainment that we observed in the short-term lottery arm after removal of the weekly lottery deadlines. The threat of learning that; “I would have had a free family vacation if I had decided differently in the past” (i.e., regret aversion) could be an explanation for this.

Regret in the future has the ability to influence health behaviors in the present by emphasizing the future consequences of current decisions [47, 48]. Results from meta-analysis by Brewer et al. [49] additionally show that the effect of anticipated inaction regret (e.g., not exercising) on health behavior is unaffected by the temporal distance of the negative consequence. Therefore, in contexts where possible regret at a distant deadline is made salient, distant deadlines may avert present-biased decision-making similarly to multiple nearby deadlines. More research on deadline distance in relation to regret would valuably contribute to the open question of the optimal duration and interval of commitment devices [22].

Scholars reviewing the effectiveness of commitment devices have concluded that the development of commitment devices is still in its early stages [22, 25, 50]. Although their design and acceptance have received considerable attention [42, 51], it remains difficult to project which contextual and behavioral features optimize its uptake and cost-effectiveness [52]. Notably, the weekly lotteries and an additional long-term lottery were effective at only about €5 per participant per week (prizes ÷ participants ÷ weeks). Because previous research has demonstrated that people are willing to put their own money at stake [25, 37] or pay premiums to restrict their future choices [42], it would be valuable to explore if and when people would also be willing to pay for lottery tickets as a commitment to their health goals.

Evidently, the costs per participant decrease if the lotteries are accepted on a larger scale. To help understand the feasibility of voluntary commitment, O’Donoghue and Rabin [17] have formalized the intuitive distinction between two extreme types of people: those who are fully aware about their future self-control difficulties (sophisticates) and those who are fully unaware (naïfs). Although both types of people may benefit from commitment devices, sophisticates are most likely to accept and profit from imposed deadlines [20, 25]. It remains unclear, however (i) if commitment devices (or meaningful deadlines) are effective if “sophisticates” accept commitment, but nonetheless have low intrinsic motivation to perform the targeted behavior and (ii) how the acceptance and use of commitment devices with a financial component may ultimately affect intrinsic motivation. Answering these open questions would valuably contribute to the effectiveness and attractiveness of commitment lotteries. Further research on the feasibility of commitment devices should focus on these unresolved questions.

Despite the financial component of the present interventions, we designed and communicated the commitment lotteries as commitment devices rather than financial incentives. Commitment lotteries differ from traditional incentives in multiple ways. First, they differ in the problem that they target. Commitment devices aim to assist people who are initially motivated to exercise on a regular basis, but believe they will probably fail to do so without proper commitment. In contrast, a financial incentive in its most traditional (neoclassical economic) sense is aimed at encouraging the unmotivated to become motivated due to the payment [53–55]. An incentive is thus a conditional cash transfer in order to increase the attractiveness of a certain behavior. In a commitment lottery, the majority of participants received no payment (approximately 84%). Besides, the expected monetary value of weekly goal attainment was low (e.g., only about €1.73 in the first 13 weeks), which is substantially lower than traditional incentives (i.e., payments) for health behavior change [37, 54, 56, 57]. Second, financial incentives differ from commitment lotteries in their contingency. In order to be eligible for a traditional cash payment, one has to perform the targeted behavior. This does not exclude a variable payment (e.g., a lottery), but traditionally, lottery *participation* is the reward for a specific health behavior [54]. In a commitment lottery, however, the imposed deadline is emphasized by the fact that all participants are included and can win, irrespective of their success. Therefore, commitment lotteries were not designed and communicated to participants as payments for attending the gym, but as a way to commit to an individual goal. Although there are multiple essential differences between a commitment lottery and a traditional lottery or simple payment, commitment lotteries also hold a clear financial component that should not be disregarded as a factor influencing the present results. For this reason, it would be valuable to explore optimal prize sizes and willingness to pay for commitment lottery tickets (hereby attenuating the financial component).

A limitation of this trial is that, although 163 participants enrolled, only six units (gyms) were randomized. Randomization at the gym level, however, avoided intervention contamination within gyms and adapted best to daily practice. That being; scientific research is not the core business of gym enterprises and researchers are safest in assuming that it has low priority in daily practice. Therefore, gyms were likely to benefit from one intervention at a time. Future research in gym contexts could extend the number of gyms.

Another limitation is that we did not directly observe PA in the gyms and assumed that participants attended their gym to exercise. An interesting topic for forthcoming research could be the effect of commitment devices on changes in the duration of gym visits or improvements in exercising routine.

Not surprisingly, sensitivity analyses showed that between-gym variation in goal attainment was highest in the control arm: in absence of a homogeneous intervention, other, non-identified factors are likely to have had more influence on goal attainment. As a result, the non-difference between the short-term arm and the control arm between Weeks 14 and 26 showed to be sensitive to exclusion of control gyms. Nonetheless, the most stringent interpretation of all results remains that the short-term lotteries are effective as long as they are present. An additional long-term deadline after weekly short-term deadlines was effective in partly preventing the decline in goal attainment after removal of the weekly deadlines.

The novel application of commitment lotteries to gym attendance has multiple benefits. First, health professionals recommend strength and endurance training 2 days a week [1], while gyms are principally equipped for this purpose. Second, offering commitment devices for gym attendance aligns with societal preferences: exercise in gyms is currently one of the most popular modes of exercise [58]. Third, reliability of PA monitoring increases as participants can only register their exercise at the gym sites. Hence, gym contexts are well suited for testing commitment lotteries, while safe and suited exercise is supervised by trained professionals [59].

Noncommunicable diseases are responsible for approximately 70% of deaths worldwide and next to significantly affecting health and well-being [60], impose a substantial economic burden [61]. Given the significant role of modifiable behavior (e.g., exercising) in preventing noncommunicable diseases and the increasing pressure on public health expenses [61, 62], there is a need for innovative low-cost approaches to health behavior change. The effectiveness of the use of personal emotions and use of social contexts [63] to support health behavior change shows promising directions in levering the impact of investments. Besides, it is not difficult to imagine possibilities for applying and further developing commitment lotteries in field settings. For example, innovative employers, governments, insurers, gyms, clinical health centers, or consortia of such could offer commitment lotteries as a part of integrated care settings. In this manner, continuous supply and reminders of voluntary deadlines for health behavior change might help avert the return to old unwanted habits [64].

## Conclusion

Many people aim to exercise on a regular basis but fail to do so. Commitment lotteries were effective in supporting regular exercise and only as long as the threat of missing out on the lottery prize was present. Weekly short-term lotteries supported regular PA for 13 weeks and an additional long-term lottery after 26 weeks showed to partly avert the decline in goal attainment after the 13 weekly lotteries. With novel applications, the current research adds to a growing body of research that shows the effectiveness of commitment devices in closing the gap between health goals and behavior.

## Notes

**Compliance with Ethical Standards Statements** The material in the manuscript has been acquired according to modern ethical standards. The trial protocol and materials were approved by the Tilburg University Ethical Review Board (EC-2014.42a). The study is registered in the Dutch Trial Register (NTR5559) and lottery drawings were performed by the independent Game Management Department of the Dutch State Lottery under supervision of a notary.

**Authors’ Statement of Conflict of Interest and Adherence to Ethical Standards** Authors Koen van der Swaluw, Mattijs S. Lambooij, Jolanda J. P. Mathijssen, Maarten Schipper, Marcel Zeelenberg, Stef Berkhout, Johan J. Polder, and Henriette M. Prast declare no conflict of interest.

**Authors’ Contributions** All people who have the right to be recognized as authors have been included on the list of authors and everyone listed as an author has made an independent material contribution to the trial.

**Ethical Approval** The trial protocol and materials were approved by the Tilburg University Ethical Review Board (EC-2014.42a).

**Informed Consent** Informed consent was obtained for all participants.
